# Effect of Cellular-Based Artificial Antigen Presenting Cells Expressing ICOSL, in T-cell Subtypes Differentiation and Activation

**DOI:** 10.34172/apb.2021.062

**Published:** 2020-08-05

**Authors:** Mehdi Talebi, Hojjatollah Nozad Charoudeh, Ali Akbar Movassaghpour Akbari, Behzad Baradaran, Tohid Kazemi

**Affiliations:** ^1^Department of Applied Cell Sciences, School of Advanced Medical Sciences, Tabriz University of Medical Sciences, Tabriz, Iran.; ^2^Immunology Research Center, Tabriz University of Medical Sciences, Tabriz, Iran.; ^3^Stem Cell Research Center, Tabriz University of Medical Sciences, Tabriz, Iran.; ^4^Hematology and Oncology Research Center, Tabriz University of Medical Sciences, Tabriz, Iran.

**Keywords:** Artificial antigen presenting cell, ICOSL, T-cell sub-types, Differentiation

## Abstract

***Purposes:*** Effective and selective T-cell activation and proliferation during the T-cell expansion phase of a cellular adoptive immunotherapy method, challenging because recent studies revealed the importance of each subtype of T-cells in different immunologic strategies against tumors, like CAR-T cell therapies. Artificial antigen presenting cells (aAPCs) regarded as a natural way to manipulate T-cell subtypes activation and specific proliferation. In the current study, we utilized K562 cells based aAPC method expressing the ICOSL molecule, to evaluate T-cell subtypes differentiation rate and functional status.

***Methods:*** CD3+T-cells isolated and, co-cultured with ICOSL expressing K562 cells. After 4, 6, and 10 days selective CD markers of T-cell subtypes and each subtype’s activity-related genes levels evaluated by qPCR methods.

***Results:*** During the culture period, CD4+ Th related phenotype reduced continuously, and in day 10^th^ of culture CD4+ T-cell’s population significantly reduced (*P* =0.029). In contrast, the CD8+ population ratio was ascending during the study period but was not statistically significant. FoxP3+CD25-, Treg population ratio was significantly increased during the time in comparison with the control group, as well as memory T-cell phenotypic marker, CD127+, expressing cells ratio. T-cell subpopulations activity-related genes expression levels evaluated too, and the Th1 related IL-2 and INF-γ reductions observed alongside regulatory T-cells gene (IL-10) and Cytotoxic T-cell’s related gene (Geranzym-A) elevations.

***Conclusion:*** We concluded that the K562-ICOSL based aAPC system is working and effective in T-cell short to medium culture periods, and this approach preparing relatively selective milieu for CD8+ T-Cell differentiation and much less Treg differentiation.

## Introduction

T-cells activation phase in adoptive immune cell therapy strategies is one of the crucial stages either for genetic manipulation, e.g. lentiviral or retroviral manipulation for chimeric antigen receptor (CAR) expression,^1^ or other relatively fewer manipulations to expansion of the cells for preparing enough amount of the cells per kilogram bodyweight of the recipients. Current methods propose utilizing natural mitogens like rhIL-2, or CD3 activating antibody (OK3) even alone or in combination in different concentrations.^2-4^ But the expansion protocol’s effectiveness, and having control on the T-cells subtypes compositions yet to be regarded.^5^ Normally T-cells composing of different subtypes with various cytokine or cell surface receptors expression profile, that cause a variety of physiological effects on the site of infection/tumor or even systematically, that finally cause the physiological balance in immunologic responses. Thus, Producing CAR T-cells from certain subtypes of CD3+ T-cells may show functional advantages,^6-8^ nevertheless in addition to importance of preparing effective T-cell proliferation strategies, having control over which subtypes of the cells may be outgrowth in a specific medium, will be an important technical tip in cellular-based technologies. Based on Hayflick’s limit theory each cell experiment relatively distinct number of cell replication, about fifty to sixty times, theoretically.^9^ This phenomenon affects cell proliferation of each T-cell subtypes, which may change the final composition of the cells prepared for genetic manipulations like CAR T-cell production, based on the unique proliferative potency of each T-cell subtype.

Physiologically T-cells activate and proliferate follow signaling by Peptide-MHC, as first signal and co-stimulatory, as the second signal, that provided by antigen presenting cells (APCs),^10-13^ the structure that provides a physical collection of these molecules, calling Immunologic Synapsis. Mature Dendritic cells (DCs) are the most effective cell-based APCs that can activate T-cells to initiate cellular immunologic responses.^14^ The other cell-based T-cell activation strategy is artificial APCs (aAPCs).^15,16^ Irradiated or fixed K562 based GMP grade aAPC systems introduced recently with satisfying efficacy^17-19^ even in T-cell activation and manipulation systems or direct use as new approaches in anti-cancer strategies,^19^ There are variety of molecules serving co-stimulatory effect on APCs but the CD28 family molecules are the most important one that can affect the kind and nature of the T-cells responses^20,21^; Of them, ICOS molecule, member of CD28 superfamily, serve specific characteristics. Despite permanent expression of CD28 on T-cells, inducible co-stimulator, express following T-cell activation.^22^ Physiologically, ICOS expression differs from tissue to tissue based on Hutloff et al, its ligation by ICOSL (also called, B7h, GL50, B7RP-1, and B7-H2) effect on T-cell proliferation is similar to CD28 mAb, but induction of IL-2 production is less than CD28 mAb.^22^

ICOSL (inducible co-stimulatory ligand, CD275) expressing on B-cells, macrophages, DCs, and some other nonlymphoid cells. Structurally, ICOSL is a single strand with two immunoglobulin-like disulfide domains, a transmembrane domain, and an intracellular domain.^23^ ICOS-ICOSL interaction shows the positive co-stimulatory effect on T-cells, as ICOS-deficient mice demonstrate marked reduction in T-cells activation and proliferation capacity and marked falling on T-cell dependent B-cell responses, marked deficiency in Ig class switching, and impaired germinal centers formation.^24-27^

Here we tried to explain the proliferation and differentiation pattern of the CD3+ T-cell of healthy donors after maximum culture time in front of ICOSL supplementation by ICOSL-expressing formalin-fixed K562 cells.

## Materials and Methods

### K562 cells preparation

ICOSL cDNA was cloned and ICOSL-K562 (KISOCL) expressing cells that were constantly overexpressing ICOSL, have been prepared by Dr. Khalafkhany D. (Duzce University, Istanbul, Turkey). ICOSL overexpression has been determined either by flow cytometry and qPCR methods (Additional file 1). The cells were fixed in 10 minutes in 0.1% formaldehyde. Cells washed and resuspended at 2 × 10^6^ /mL by PBS+BSA (5%) and transferred to our lab. The fixation protocols have been done based on previous Tanimoto et al work.^18^

WT K562 cells were cultured at RPMI-1640 supplemented by 10% FBS and 100 IU/mL penicillin and 100 ug/mL streptomycin, at 37°C and 5% Co2. And after the expansion period, the cells washed by PBS+ BSA (5 g/L) formulated as 2 × 10^6^ cells/mL and fixed with 0.1% formaldehyde at the final concentration for 10-20 minutes.

### T-cell Isolation and culture

T-cells were collected from leukapheresis sterile samples prepared from allogenic Bone marrow donors sample referred to Shahid Ghazi Hospital Laboratory for CD34 and CD3 counting. Donors had been conditioned by subcutaneous G-CSF, 5-7days before cytapheresis. Brief, 100 μL PBMC sample collected from healthy Bone Marrow Donors incubated with 5 μL anti-CD3-FITC (BD, USA) antibody in darkness for 45mins and washed by sterile PBS and resuspended in PBS-5%BSA solution. Then the CD3+ cells gated on lymphocytes region and approximately 0.5 × 10^6^-1 × 10^6^ cells sorted by the FACSCalibur Flowcytometry system (BD, USA). The cells centrifuged immediately after sorting in 4°C and transferred to 10 mL RPMI-1640+15%FBS with 100 IU/mL Penicillin/ 100ug/ml streptomycin and 2 mmol/L L-glutamine medium and 2-5 ug/mL phytohemagglutinin (PHA) (Gipco, Germany). Cell viability evaluated by Trypan blue dye exclusion method and formulated as 10^4^/mL cells for next culturing steps.

In 12 well culture dishes, approximately 10^4^ cell/cm^2^ seeded and grouped in three; Control, Wild type fixed K562 (KWT), and ICOSL expressing fixed K562 cells (KICOSL) co-cultured with T-cells. Fixed WT-K562 used in the same way in the control group.

Cells counted and viability checked by Trepan blue Exclusion method at first, 3^rd^and 7^th^ days of culture time. After counting both WT or ICOSL-K562 cells were entered to media in 100:1 effector target ratio of each day of culture time and the cells cultured for three additional days. After that the cells harvested (1^st^ at 4^th^ day, 3^rd^ at 6^th^ day and 7^th^ at 10^th^ day of initial culture time) and divided for RNA extraction and flow cytometry analysis.

### Flow cytometry analysis

Key T-cell subtypes surface markers and activity markers relative expression levels evaluated by flow cytometry method.

At 4^th^, 6^th^, 10^th^ days the cells harvested from each concentration group, 200-500 × 10^3^ cells prepared for CD markers analyzing. CD4-FITC (as Helper T marker), CD8-PE (as Cytotoxic T marker), CD25-PE, FOXP3-PerCp (as regulatory T-cell marker together with CD4) and CD127-FITC (as memory differentiation of T-cells) (BD, Bioscience, USA) based on single or triple panel orders. Briefly 3-5 μL of respective antibody-incubated for 45 minutes to one hour with cells in darkness and washed by PBS, the pellet resuspended in 500-1000 μL PBS and analyzed by BD-FACSCalibur flowcytometry systems. For intracellular markers, permeabilization steps before antibody steps have been done based on manufacturer instructions.

### RNA isolation and gene expression analysis

The second portion of the harvested cell’s (approximately 0.5×10^6^-1×10^6^/mL cell) RNA extracted by the Cinnagen RNAX-plus RNA extraction kit (Sinnaclone, Iran) and cDNA produced by Smobio (China) cDNA synthesis kit, based on manufactures instruction. The qPCR performed by Corbette (Rotorgene, Thermo, Germany) system. Selected genes are the major genes related to different T-cell subtypes properties and/or activation status. PCR done in duplicate and reactions were performed in 20 μL final volume, with 0.5 μL forward and reverse primers (10 pmol), 10 μL SYBER Green PCR master mix, 3 μL cDNA, and deionized water to final volume. The genes and primers list mentioned in Table 1. Generally, the PCR thermal cycles were as 95°C for 15 minutes as initial denaturation phase following 95°C (35”), 65°C (25’), 72 °C (30”) for 40 cycles. The Data have been analyzed by relative fold-change in expression level (RFC) (2-^ΔΔCt^) method.

**Table 1:** Primers list used for important immunological response genes expression

**Gene Name**		**Primers Sequences**
IL-2	F	ACCAGGATGCTCACATTTAAGTTTT
	R	GAGGTTTGAGTTCTTCTTCTAGACACTG
IL-10	F	GCCGTGGAGCAGGTGAAG
	R	GAAGATGTCAAACTCACTCACTCATGGCT
IFNg	F	AGCTCTGCATCGTTTTGGGTT
	R	GTTCCATTATCCGCTACATCTGAA
TGF-β1	F	CGAGAAGCGGTACCTGAAC
	R	TGAGGTATCGCCAGGAATTGT
GZMA	F	GGGACGATGTGAAACCAGGA
	R	AGGCTTCCAGCACAAACCAT
B-actin	F	GGCACCCAGCACAATGAAG
	R	GCCGATCCACACGGAGTACT

### Statistical analyzing

The descriptive data expressed as frequency and mean ± SD, and for parametric data, an independent sample t-test performed. For comparisons between groups, two-way ANOVA following Bonferroni multiple comparison tests have been performed, and for non-parametric or nominal parameters χ^2^ analysis performed by GraphPad Prism version 8.0.2 (GraphPad Software, Inc., La Jolla, CA, USA), *P* value<0.05 regarded as statistically significant.

## Results

### Cell surface markers expression

**Figure 1:**
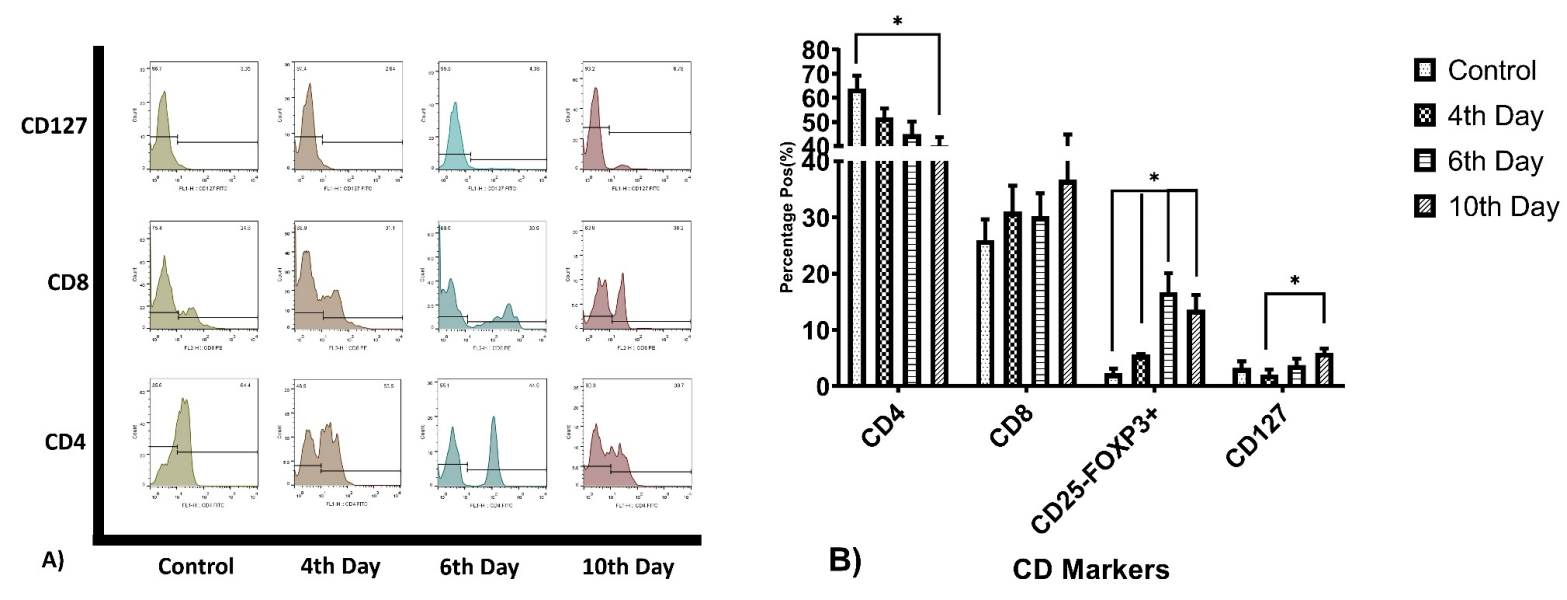
T-cell subtypes Related CD markers expression percentage. **(A)** represents CD4, CD8, and CD127 expression ratio on T-cells, because CD25-FoxP3+ subtype calculated and not show on the figure. **(B)** the bar char summarized the relative expression of the Markers. Th related CD4 expression gradually reducing during the culture period, and Tc marker and Treg and memory T-cells percentage gradually increasing (*P value<0.05).

CD3+ T-cells have been evaluated for CD4, CD8, CD127, and FoxP3+CD25- cells calculated from relative dot-plot data. The mean percentage of CD4+ cells in the control group was 64±5.3%, at day 4^th^ was 52±3.68, 6^th^ day 45±5.3 and at 10^th^ day the mean percentage was 40.30±3.5. The CD8+ cell population for these days was 26±3.69, 31.03±4.63, 30.2±4.08, and 36.7±8.20. The full data of T-cell subpopulations major CD markers frequencies summarized in Table 2. The analysis revealed that CD4+ cells population ratio significantly reduced at day 10, by 23.7% (*P*=0.034) and CD25-FoxP3+ cell population raised from 2.3% to 13.6% (*P* = 0.029), by the way, the elevation on this population’s percentage during the period of study was relatively continuous in compare with the control group, Figure 1. During this period there was no significant variation observed in other cell population frequency.

**Table 2:** T-cell major subpopuaclation related markers frequency (mean%±SD)

**CD Marker**	**Control**	**4th Day**	**6th Day**	**10th Day**
CD4+	64±5.3	52±3.68	45±5.3	40.3±3.5
CD8+	26±3.69	31.03±4.63	30.2±4.08	36.7±8.2
CD25-FOXP3+	2.3±0.86	5.62±0.15	16.73±3.41	13.6±2.65
CD127+	3.24±1.2	2.1±0.84	3.78±1.1	5.98±0.73

### Immunologically important genes expression

**Figure 2:**
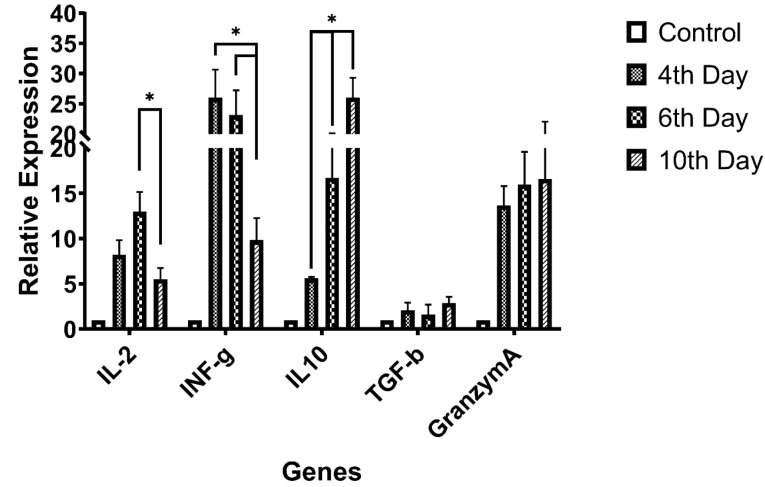
Selective genes expression for T-cell subtypes activity (differentiation),IL-2, and INF-γ genes relative expression reducing during culture period but regulatory cytokine gene expression (IL-10) and cytotoxic T-cell activity protein Granzyme-A level was increasing (*P value<0.05).

Selective genes relative expression status, assessed and the data have been summarized in Figure 2. The expression levels have been normalized to control group expression status and reported as relative expression ± SD. According to data, there was a significant fall in IL-2 gene expression between day 6^th^ and 10^th^ observed (*P* = 0.048), the INF-γ gene expression was in continuously reductive state during the time (*P* = 0.041). Granzyme-A and TGF-β genes expression rates were relatively ascending but data were not statistically significant (*P* > 0.05).

## Discussion

Despite the importance of the rapid effective T-cell expansion and activation in adoptive immune-cell therapy methods, either for virus transduction improvement and or harvesting enough cell count for transplantation, also the slope or rate of the activation status good to be regarded in these strategies. Utilizing cell-based aAPCs as an effective way for T-cell activation have been introduced in previous studies, by this mean, a technologist can expand the distinct subtypes of the T-cell to maximizing desired immune response modality. As some studies show that, expanding and transplantation of some subtypes of T-cell will show effective tumor elimination phenomenon, for example, Berger et. al. showed that clonally derived CD8+ T-cells isolated from central memory T-cells are distinct from those derived from effector memory T-cells and retain an intrinsic capacity that enables them to survive after adoptive transfer and revert to the memory cell pool.^7^ Moeller and colleague showed that CD4+ T-cells manipulated to express lung cancer-specific antigens specificity, more effectively eliminate tumor cells than non-selected lymphocyte mixes.^28^ It has been shown that ICOSL-ICOS interaction is necessary for T-cell activation,^27,29^ the point that not paid attention in current T-cell activation and proliferation protocols. T-cell activation by this co-stimulatory pathway causes T-cell proliferation without increasing in IL-2 secretion levels,^30^ our finding showing that, IL-2 elevation from first to 6^th^ day was ascending but it was not statistically significant. Also, a significant reduction in IL-2 on the 10^th^ day can be attributed to cell exhaustion, the phenomenon that not examined in this study. In this study, we tried to avoid using rhIL-2 or OK3 to prepare the natural condition as much as possible. But this condition may be so exhaustive for the cells than conventional methods, as expected. About INF-γ, another major cytokine of Th1 subtypes, lymphocytes cultivation with ICOSL-K562 cells demonstrates a significant elevation in 4^th^ day, in gradually reduces till day 10^th^. In contrast expression patterns of the IL-10 and TGF-β were ascending by time, which altogether with previous data reveals more regulative differentiation of T-cells. Despite the regulatory differentiation of the cells, Cytotoxicity marker’s levels elevation together with CD8+ cells increasing, Figure 1, revealed CD8+ T-cell differentiation by supplementing culture by CD275 expressing cells. We observed graduate increasing in CD127+ T-cells but not completely statistically significant, except 4^th^ and 10^th^ days, it revealed memory T-cell differentiation but in less efficacy than other reports have been shown by Mahajan et. al. or Burmeister and colleague’s reports.^31,32^

## Conclusion

Accordingly, we concluded that the K562-ICOSL based aAPC system is working and effective in T-cell short to medium culture periods, and this approach preparing relatively selective milieu for CD8+ T-Cell differentiation and much less Treg differentiation. But as the effective Treg differentiation and activation need more complex systems, and CD25+FoxP3+ cell’s effects have not been included in this study, more data will be needed to decide whether Regulatory differentiation in presence of K562-ICOSL cells are functional or not.

## Ethical Issues

All the research processes were ethically certified by Iran national committee for Ethics in biomedical Researches as IR.TBZMED.REC.1397.611.

## Conflict of Interest

Authors declare no financial or non-financial conflicts of interest.

## Acknowledgments

This study was granted by Tabriz University of Medical Sciences, Research Vice-Chancellor, Faculty of Advanced Medical Sciences of Tabriz University of Medical Sciences, and Immunology Research Center, Tabriz University of Medical Sciences (Project No.: 60321).
